# Atrial tachycardia occurring after a prior atrial fibrillation ablation procedure: Does non-inducibility matter?

**DOI:** 10.3389/fcvm.2022.1073239

**Published:** 2022-12-07

**Authors:** Louisa O’Neill, Benjamin De Becker, Maarten De Smet, Jean-Benoit Le Polain De Waroux, Rene Tavernier, Mattias Duytschaever, Sebastien Knecht

**Affiliations:** Department of Cardiology, AZ Sint-Jan Hospital, Bruges, Belgium

**Keywords:** atrial tachycardia (AT), atrial tachycardia ablation, inducibility, linear ablation, outcomes, vein of Marshall ablation

## Abstract

Recurrent atrial tachycardia (AT) is a common phenomenon after catheter ablation for AF, particularly in the setting of additional substrate ablation, with many studies demonstrating gap-related macro re-entrant AT (predominantly mitral and roof dependent) to be the dominant mechanism. Although multiple inducible ATs after ablation of the clinical AT are commonly described at repeat procedures, the optimal ablation strategy, and procedural endpoints are unclear in this setting. A recent randomized study addressing the question of non-inducibility as a procedural endpoint demonstrated no additional benefits to the ablation of all induced, non-clinical ATs, but it was limited by small numbers and high rates of non-inducibility. Nevertheless, once ablation of the clinical AT has been successfully performed, ensuring durable linear block and PV isolation may be sufficient for the prevention of further AT. Durable linear block, particularly at the mitral isthmus, is difficult to achieve but may be facilitated by the real-time evaluation of lesion quality and contiguity and the novel technique of vein of Marshall ethanol infusion. Large-scale, randomized trials are needed, nonetheless, to fully assess the optimal ablation strategy in the setting of recurrent AT post-AF ablation.

## Introduction

Atrial fibrillation (AF) represents the most common cardiac arrhythmia worldwide. Catheter ablation is a cornerstone therapy and carries a class I indication for the management of symptomatic drug-resistant, paroxysmal, and persistent AF (1). Advances in catheter and mapping technology over the last decade have been reflected in greater numbers referred for ablation, and a related consequence is the increased incidence of atrial tachycardia (AT) post-index procedure, which varies from 5 to 40% in the literature (2). These tachycardias tend to be incessant, poorly tolerated, and unresponsive to pharmacological agents (3, 4). Given the often-continuous nature of arrhythmia, catheter ablation is the treatment of choice and is facilitated by high-density mapping systems. An important issue with repeat ablation for recurrent AT is that of further inducible ATs after the ablation of clinical AT. The majority of studies evaluating recurrent AT after index AF ablation tend to target these additional inducible ATs for ablation and use non-inducibility as a procedural endpoint (5–11). Nevertheless, the evidence to support this strategy is scanty with a paucity of studies evaluating the value of inducibility testing and indeed the prognostic implications of persistent, inducible AT at the procedural end. While the elimination of all inducible ATs seems reasonable, it may serve only to prolong procedure times and to create a further substrate for recurrent arrhythmias. In this mini-review, we outline the literature regarding the ablation of AT post-AF ablation, specifically in relation to multiple inducible ATs and procedural endpoints.

## Type, prevalence, and management of recurrent atrial tachycardia post ablation

The widespread adoption of catheter ablation for AF coupled with the advent of novel single-shot technologies may result in increased numbers undergoing redo procedures for recurrent AT in the coming years. Ablation strategy at index procedure plays a key role in the likelihood of developing post-procedure AT. With pulmonary vein isolation (PVI) alone, AT has been reported at <5% and may relate to PV reconduction, with focal ATs often described in the setting of earlier studies of segmental or ostial PVI (12, 13). The incidence of AT rises in the setting of additional ablation beyond PVI, increasing left atrial size and non-paroxysmal AF (14) with macro re-entrant ATs predominating (8), most commonly mitral isthmus and roof-dependent (7). More recently, recurrent AT has been described with new “single-shot” techniques, including cryoballoon PVI, and again frequently takes the form of macro re-entrant tachycardia (10, 11). Additionally, micro re-entrant ATs have been described in zones of previous ablation and slow conduction (15) and may be associated with extensive prior ablation. Although the guidelines are clear regarding the role of index catheter ablation for AF, there are no current recommendations with respect to the indication for, or ablation strategy during redo AT ablation. The 2019 ESC guidelines for the management of supraventricular tachycardia suggest that intervention for recurrent AT should be delayed for at least 3 months post-AF ablation procedure, and that pharmacological rate or rhythm control may be preferable initially but makes no formal recommendations on the subject (16).

In real-world practice, ablation strategy during redo AT ablation is facilitated by high-density mapping systems, allowing for accurate determination of AF mechanisms and critical isthmuses. Although acute procedural success rates of up to 85% have been previously described, with high rates of termination of focal and micro re-entrant ATs in particular (7), recurrence rates of ∼30% are reported (7, 17). In particular, macro re-entrant ATs tend to recur, most commonly peri-mitral flutter (18). This highlights the need for the optimization of procedural workflows and a better understanding of appropriate endpoints.

## Atrial tachycardia non-inducibility as a procedural endpoint: Evidence and current practice

As aforementioned, in many patients with recurrent AT after AF ablation, multiple ATs beyond the clinical AT were seen at the time of the redo procedure. Chae et al. mapped 155 ATs in 78 patients undergoing repeat procedures after prior PVI (7), while multiple ATs were successfully characterized in >50% of patients in a subsequent prospective cohort of 128 patients post-AF ablation (19). Furthermore, Rostock et al. reported an average of 2.2 ATs per patient in a similar population (20). It appears to be a widely accepted practice to attempt to induce further ATs after ablation of the clinical AT and to target these for ablation until non-inducibility (5, 6, 8, 10, 21). Inducibility protocols that are frequently described include rapid atrial pacing to atrial refractoriness and programmed atrial stimulation with or without the use of isoprenaline (7–10). While ablation of all ATs to non-inducibility may be considered a reasonable procedural endpoint, data are lacking regarding the benefits of further ablation beyond the clinical tachycardia and the prognostic implications of such additional inducible ATs. Although programmed atrial stimulation for inducing AT may be useful in patients with documented arrhythmia, it is not a highly sensitive or specific technique (22, 23). Furthermore, studies evaluating the prognostic significance of inducible AT were predominantly performed at the time of AF ablation rather than during the repeat procedure for recurrent AT. In 2005 Chugh et al. (9) reported an association between spontaneous or induced AT seen after ablation for AF and recurrent AT on follow-up. Nevertheless, only a small proportion underwent a repeat ablation for AT in this study. More recently, a non-randomized study by Nagamoto et al. reported rates of inducible AT of >50% in patients undergoing PVI and substrate ablation (24). While there was no overall difference in outcome according to inducibility status, recurrent AT was lower in those in whom inducible ATs were successfully ablated compared to those still inducible at the procedural end. Nevertheless, recurrent AT on follow-up tended to be different from that induced at the time of the index procedure, questioning the overall relevance of inducible AT post-AF ablation. In 2018 Santangeli et al. (25) reported on 305, predominantly paroxysmal, patients with AF undergoing an induction protocol before and immediately after AF ablation. They described a 39% rate of inducible AT or AF post-ablation with no relationship between inducibility status and outcome. Unlike the study by Nagamoto et al., however, no inducible ATs were targeted for ablation in this study. Similarly, in 2019 Kawai et al. (26) found no association between non-ablated inducible AT (or AF) after persistent AF ablation and outcomes, except in those with left atrial size enlargement on sub-analysis.

## Prognostic implications of atrial tachycardia inducibility

**FIGURE 1:**
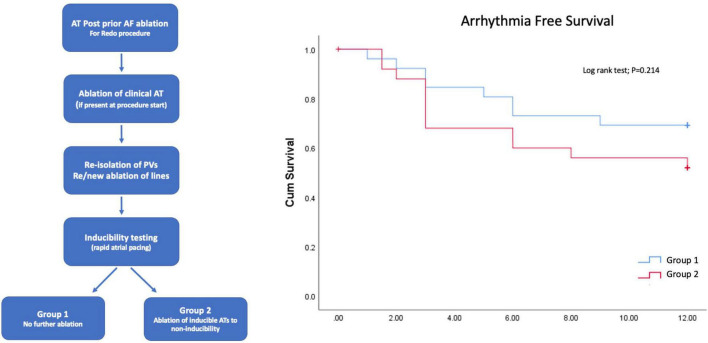
Study design **(left panel)** and results **(right panel)** from the randomized Inducath study. No difference in arrhythmia-free survival was seen between those undergoing ablation of inducible atrial tachycardias (ATs) to non-inducibility compared to those undergoing ablation of the clinical AT only.

Overall, the above studies do not appear to suggest a benefit from inducibility testing at the time of AF ablation and do not address the issue at the time of the repeat procedure for clinically relevant AT. A small, recently published, randomized study (Inducath) attempted to bridge this knowledge gap and answer the question of whether ablation of all inducible ATs to non-inducibility, during repeat AT ablation, would improve outcomes (27). In total, 52 patients with recurrent AT post first-time ablation for persistent AF were randomized into either a conservative group, in whom ablation of the clinical AT only was performed in addition to re-isolation of the PVs and re- or new ablation of lines (with confirmed block) or a “non-inducibility” group, who underwent this strategy with additional ablation of further inducible ATs (Figure 1). The inducibility protocol consisted of repetitive atrial burst pacing to a minimum rate of 200 ms. In line with prior reports, the majority of clinical ATs were peri-mitral re-entries. Interestingly, after ablation of the clinical AT and re-ablation of prior lesion sets, a high rate of non-inducibility was seen with inducible AT in 19 vs. 35% in either group (*P* = n/s). There was no difference in arrhythmia-free survival at 1 year between those with vs. without further inducible AT nor did non-inducibility at the procedural end affect the outcome. Furthermore, in those patients with inducible AT, there was no difference in outcome between those undergoing ablation to non-inducibility vs. a conservative ablation strategy (Figure 1). A major limitation of the study was its small size, which, coupled with the low rate of non-inducibility limited the number of patients undergoing the protocol-mandated ablation strategy.

Bearing in mind its limitations, the results of the Inducath study suggest that achieving long-term durability of both the PV and linear lesion sets may be of greater value than ablating all non-clinical ATs to non-inducibility in the future prevention of AT. Low rates of additional inducible AT may reflect the effect of ablation of the clinical AT on the underlying arrhythmia substrate and importantly the re-ablation of prior lines and PVs (in those demonstrating recovered conduction) prior to the induction protocol, potentially eliminating additional critical isthmuses for AT.

A recent study by Takigawa et al. reported on AT mechanisms at first and second repeat AT ablation after initial AF ablation (18). Consistent with prior literature, macro re-entrant mitral and roof-dependent AT were predominant mechanisms. Despite effective termination of the clinical AT and even with subsequent non-inducibility, recurrence occurred in 38%, again with a predominance of mitral followed by roof-dependent AT. Conversely, while localized ATs also tended to recur, they were not of the same mechanism at redo vs. initial AT procedure. Local re-entry is commonly described in the setting of prior extensive ablation, and ablation strategies for these ATs are aimed at eliminating areas of fractionation, indicating slow conduction in low-voltage regions (28). Given the results of the above-mentioned two studies, it may be the case that rather than a procedural target, further inducible ATs of this nature may merely represent a marker of more advanced arrhythmogenic remodeling. Indeed, ablation of these sites without linking to an anatomical structure may increase the risk of further recurrences. Overall, these studies would suggest that durable mitral isthmus and roof block are the key determinants of a successful outcome in AT ablation of this nature.

## Importance of durable linear lesion sets

Regarding the effect of linear ablation during AF ablation on the risk of developing recurrent AT, conflicting reports exist in the literature. A randomized study in 2004 demonstrated a reduced incidence of AT when linear roof and mitral ablation was performed in addition to PVI during AF ablation (29), findings echoed by Knecht et al. (30). Conversely, several subsequent studies demonstrated increased rates of macro re-entrant AT with linear ablation beyond PVI (8, 14). When performing lines at the index AF procedure, the importance of durability is undisputed, however, with much evidence to support the presence of gaps as critical to the development of recurrent macro re-entrant AT (7, 30–32). Indeed, in prior studies, up to 90% of recurrent AT have been demonstrated to be gap-related (7), with up to 60% described as having a critical isthmus at the mitral isthmus (9). In recent years, improvements in catheter and ablation technology have focused on lesion set durability at the index procedure. To this end, protocols employing contiguous lesions with targeted “ablation index” values have translated into robust acute and long-term success rates for PVI with high rates of durability seen at repeat procedure (33–35). With respect to linear ablation, the ALINE study examined the effect of similarly optimized, contiguous RF lesion delivery on the rate of first-pass block at the left atrial roof and mitral isthmus and reported a high rate of first-pass block at the roof but not the mitral line using this protocol (36). Additional endocardial and epicardial applications resulted in a final rate of bidirectional mitral line block of 80%, emphasizing the challenges with RF ablation alone at this site. In the above-mentioned study of AT mechanisms by Takigawa et al., epicardial structures were involved in 75% of mitral macro re-entrant circuits, predominantly the coronary sinus and vein of Marshall (VoM) system, with a lesser proportion of roof-dependent ATs also utilizing epicardial structures (18). This and the predominance of peri-mitral and roof flutters seen post-AF ablation again underscore the importance of durable linear block and highlight the difficulty of achieving this on the long term, particularly at the mitral isthmus.

## Epicardial connections and novel techniques

Most notably in the case of the complex three-dimensional anatomy of the mitral isthmus, endocardial block may be difficult to achieve, with gaps frequently resulting from coronary sinus and VoM epicardial connections (18). The latter is electrically insulated from the left atrial myocardium by adipose tissue (37), which may further explain the low rate of block achieved with endocardial ablation alone. As such ethanol infusion of VoM was developed as an adjunct to RF ablation, with recent work indicating more durable block at repeat procedure and less RF ablation (endocardially and in the coronary sinus) needed to achieve acute intraprocedural block (38–40). Furthermore, a randomized study demonstrated reduced rates of recurrent AT (and AF) on follow-up in those receiving adjunct VoM ethanol infusion during ablation for persistent AF (41), with peri-mitral block identified as a significant determinant of outcome (42). A recently published meta-analysis of the technique confirmed these findings with greater freedom from recurrent AT and AF with adjunct VoM ethanol infusion compared with ablation alone in patients with AF (43). Epicardial connections across roof lines utilizing the septopulmonary bundle, which again may be insulated by fat, have been demonstrated to be a common cause of failure to achieve roof line block (44). In this setting, a floor line may be appropriate and is associated with high rates of transmural block.

## Conclusion

In the case of recurrent AT after index AF ablation, once ablation of the clinical tachycardia has been performed and linear block confirmed, the prognostic value of AT inducibility testing and the use of non-inducibility as a procedural endpoint appears questionable. While macro re-entrant roof and peri-mitral tachycardias tend to recur at repeat procedure, recurrent localized ATs can be different in mechanism from those seen at the initial AT procedure. Indeed, ablating the clinical AT alone and ensuring durable linear block and PV isolation may be the optimal strategy in this setting. Further ablation of inducible ATs may serve only to prolong procedure times and create an additional substrate for recurrent arrhythmia, although this still warrants evaluation in large-scale trials.

Given the high rate of peri-mitral flutters seen in prior studies, obtaining persistent block at the mitral isthmus may represent one of the most important factors in the long-term maintenance of sinus rhythm but is difficult to achieve with endocardial ablation alone. VoM ethanol infusion demonstrates promise for facilitating durable mitral isthmus block and preventing recurrent AT, but needs ongoing assessment in prospective clinical trials.

## Author contributions

All authors listed have made a substantial, direct, and intellectual contribution to the work, and approved it for publication.
